# LPS-Activated Microglial Cell-Derived Conditioned Medium Protects HT22 Neuronal Cells against Glutamate-Induced Ferroptosis

**DOI:** 10.3390/ijms24032910

**Published:** 2023-02-02

**Authors:** Mauricio Tavares Jacques, Luciano Saso, Marcelo Farina

**Affiliations:** 1 Department of Biochemistry, Federal University of Santa Catarina, Florianópolis 88040-900, Brazil; 2 Department of Physiology and Pharmacology “Vittorio Erspamer”, Sapienza University of Rome, 00185 Rome, Italy

**Keywords:** BV-2, HT22, conditioned media, glutamate, tert-butyl hydroperoxide, ferroptosis

## Abstract

Neuron-glia interactions are essential for the central nervous system’s homeostasis. Microglial cells are one of the key support cells in the brain that respond to disruptions in such homeostasis. Although their participation in neuroinflammation is well known, studies investigating their role in ferroptosis, an iron-dependent form of nonapoptotic cell death, are lacking. To address this issue, we explored whether microglial (BV-2 cells) activation products can intensify, mitigate or block oxidative and/or ferroptotic damage in neuronal cells (HT22 cell line). Cultured BV-2 microglial cells were stimulated with 5–100 ng/mL lipopolysaccharide (LPS) for 24 h and, after confirmation of microglial activation, their culture medium (conditioned media; CM) was transferred to neuronal cells, which was subsequently (6 h later) exposed to glutamate or *tert*-butyl hydroperoxide (*t*-BuOOH). As a major finding, HT22 cells pretreated for 6 h with CM exhibited a significant ferroptosis-resistant phenotype characterized by decreased sensitivity to glutamate (15 mM)-induced cytotoxicity. However, no significant protective effects of LPS-activated microglial cell-derived CM were observed in *t*-BuOOH (30 µM)-challenged cells. In summary, activated microglia-derived molecules may protect neuronal cells against ferroptosis. The phenomenon observed in this work highlights the beneficial relationship between microglia and neurons, highlighting new possibilities for the control of ferroptosis.

## 1. Introduction

The brain exhibits a high requirement for oxidative metabolism. Indeed, approximately 20% of the oxygen used by the human body at rest is designated for cerebral functions [1]. Such high oxygen consumption leads, consequently, to a higher production of oxygen-derived reactive and oxidant species, which, summed to a modest brain’s antioxidant defenses, render this organ highly susceptible to oxidative stress [2]. Of particular importance, neurons exhibit a higher rate of oxidative metabolism [3] and lower antioxidant capacity [4] compared to glial cells, which makes this cell type highly prone to oxidation. Additionally, neuronal membranes can be composed of polyunsaturated fatty acids (PUFA), which are sensitive to the action of oxidants [5]. In this scenario, neurons are highly susceptible to oxidative damage and degeneration, which seem to play pivotal roles in the development of neurodegenerative diseases, such as Parkinson’s, Alzheimer’s and amyotrophic lateral sclerosis [5,6,7,8]. Among the pathophysiological events underpinning the development of such neurodegenerative diseases, excitatory amino acid toxicity, impaired energy metabolism and oxidative stress represents major events [9]. Of particular importance, a recent form of nonapoptotic programed cell death, called ferroptosis, has emerged as a pathway of interest in the aforementioned context [10].

Ferroptosis, a recently described form of regulated nonapoptotic cell death, is characterized by a loss of control over iron metabolism, thiol regulation and lipid peroxidation [11]. It also has hallmarks such as glutathione (GSH) depletion, glutathione peroxidase 4 (GPX4) disruption, increased levels of reactive oxygen species (ROS) and intense lipid peroxidation [12]. Interestingly, features present in ferroptosis, such as GSH depletion, lipid peroxidation, mitochondrial dysfunction and Ca^2+^ dysregulation, are also present in aging, Alzheimer’s and Parkinson’s diseases, amyotrophic lateral sclerosis and stroke, suggesting a possible participation of this pathway in these conditions [13]. Therefore, mitigating or blocking ferroptosis in neurons (and brain environment) becomes a plausible therapeutic target of interest. This possibility can be supported by different approaches, which aim, for example, to reduce local oxidative stress by supporting antioxidant defenses or directly acting on reactive species. Despite the well-known supportive metabolic and antioxidant roles of astrocytes toward neurons [14], studies on the relationship between glial cells (including microglia) and neurons in the ferroptotic context are absent.

Microglia are immune cells resident in the central nervous system, described as double-edged swords, as they have protective or damaging aspects, improving or aggravating the physiological or pathological condition in which they act [15]. The dysregulation of the sentinel, housekeeping and defense functions of the microglia is associated with neuronal damage in diseases such as amyotrophic lateral sclerosis, Alzheimer’s, Parkinson’s, Huntington’s and stroke [15,16]. Although an “activated” microglia is capable of releasing numerous factors that can damage neurons, we cannot disregard the possible protective role of these signals, since there is a complex cross-talk between microglia and neurons and the result of these interactions could be beneficial in certain conditions [17,18,19]. However, as already mentioned, studies on the relationship between microglial cells and neurons in the ferroptotic context are lacking.

Considering that (i) neurons are particularly sensitive to oxidative damage, (ii) ferroptosis seems to play a significant role in neurodegenerative conditions and (iii) microglial cells (mainly when activated) produce a variety of molecules capable of directly and indirectly affect neurons, we investigated whether the conditioned media of cultured microglia stimulated with lipopolysaccharide (LPS, a classic microglial stimulator) would be able to provide modulatory (protective or deleterious) effects in neuronal cells (HT22 cell line) challenged by models of oxidative toxicity—glutamate-induced ferroptosis and *tert*-butyl hydroperoxide (*t-*BuOOH)-mediated oxidative damage. We observed that the conditioned medium derived from LPS-stimulated microglia prevented glutamate- (but not *t-*BuOOH) induced cytotoxicity. This observation showed the protective effects of microglia-derived molecules against neuronal ferroptosis, shedding light on a new event related to the well-known beneficial relationship between neuronal and glial cells.

## 2. Results

### 2.1. LPS Is Not Cytotoxic and Induces Microglial Activation

**Figure 1:**
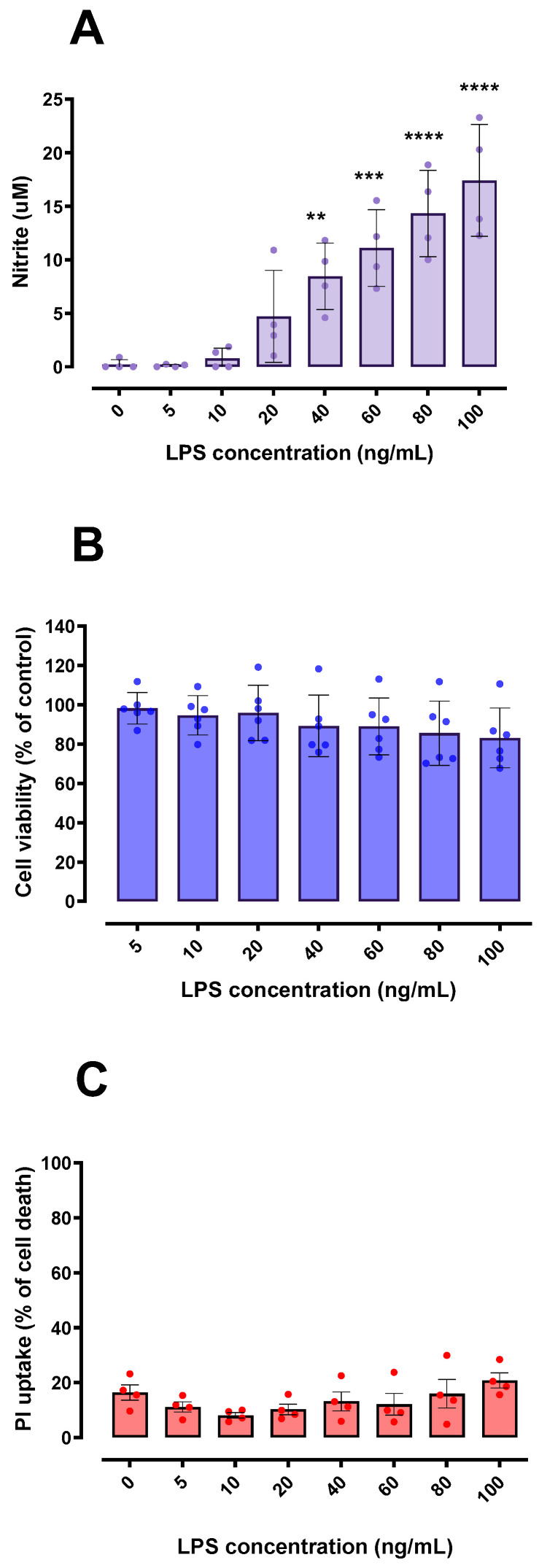
Effects of LPS on nitrite release and cell viability in BV-2 microglial cells. Concentration-dependent nitrite release from BV-2 cells was detected by the Griess method, suggesting microglial activation (**A**). BV-2 cell viability was not significantly affected by LPS exposure when compared to the control using MTT and PI assays (**B**,**C**). In (**C**), 100% cell death is represented by cells treated with 0.2% Triton X-100 for 20 min. ** *p* < 0.01; *** *p* < 0.001; **** *p* < 0.0001 in comparison with control group (untreated cells). (**A**,**C**) *n* = 4; (**B**) *n* = 6.

BV-2 microglial cells were responsive to different concentrations of LPS, with significant increased production of nitrite from 40 ng/mL of LPS (Figure 1A). Nitrite is a stable end-product of nitric oxide (NO) metabolism, used as a proxy to indicate microglial activation [20]. Even though the higher LPS concentration (100 ng/mL) caused ≅ 15% reduction in the BV-2 cells’ capability to reduce MTT (Figure 1B), LPS effect was not statistically significant [F (6, 35) = 0.99; *p* = 0.44]. Thus, we assumed that the concentrations of LPS used, which were able to stimulate BV-2 cells, did not cause major changes in cell viability (Figure 1B,C) and were therefore used in this work as a standardized approach to obtain the conditioned media (CM).

### 2.2. LPS and CM Were Not Cytotoxic in HT22 Cells

**Figure 2:**
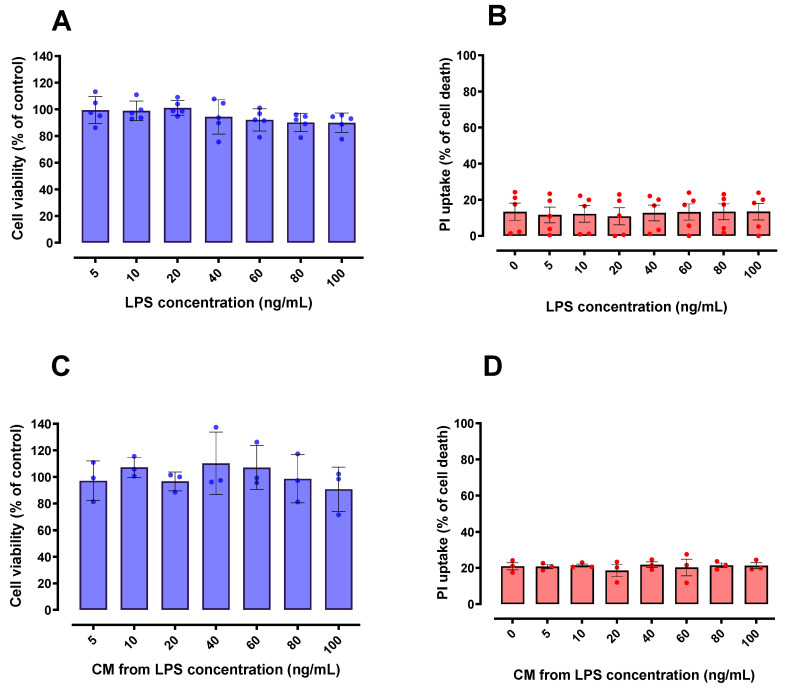
Effect of LPS and conditioned media (CM) on HT22 cell viability. Different concentrations of LPS (**A**,**B**) and CM produced by LPS-stimulated BV-2 cells (**C**,**D**) did not significantly affect cell viability of HT22 cells when compared to control group (untreated cells); 100% death: cells treated with 0.2% Triton X-100 for 20 min. *n* = 5 (**A**,**B**); *n* = 3 (**C**,**D**).

In order to initially discard potential neurotoxic effects of LPS and/or CM derived from LPS-stimulated BV-2 cells, the cultured HT22 cells were exposed to both potential challenges. We found that HT22 cell viability was not compromised by LPS (Figure 2A,B) or by the CM derived from LPS-activated BV-2 cells (Figure 2C,D). From a methodological and experimental point of view, these results were necessary to discard the occurrence of direct cytotoxic effects of LPS and/or LPS-derived CM in HT22 cells, and to further explore the potential beneficial effects of such CM in this neuronal cell line.

### 2.3. Glutamate and t-BuOOH Induced a Concentration-Dependent Cytotoxicity in HT22 Cells

**Figure 3:**
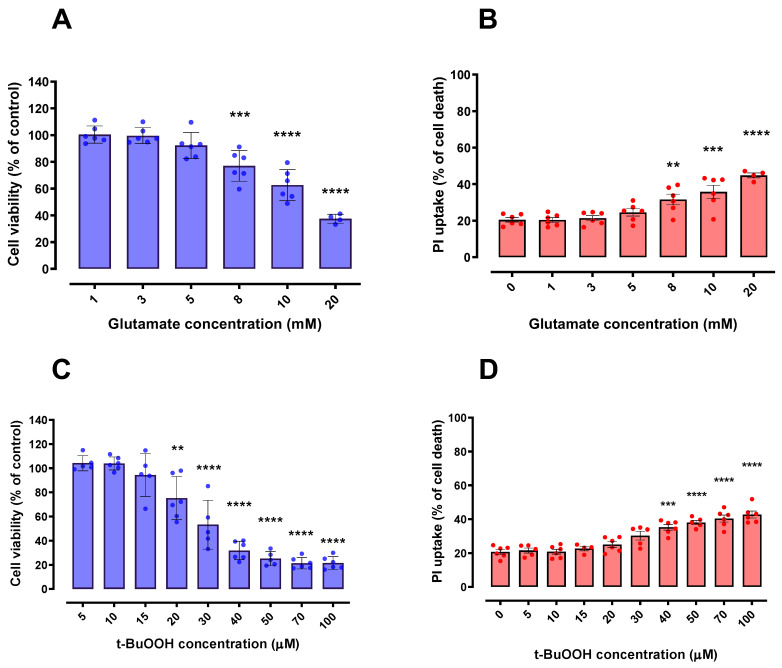
Effects of Glutamate and *t*-BuOOH on HT22 cell viability. HT22 cells exposed to glutamate (**A**,**B**) and *t*-BuOOH (**C**,**D**) exhibited a progressive (concentration-dependent) impairment of cell viability in both tested methodologies (MTT and PI). In (**B**,**D**), 100% cell death is represented by cells treated with 0.2% Triton X-100 for 20 min. ** *p* < 0.01; *** *p* < 0.001; **** *p* < 0.0001 compared to control group (untreated cells). *n* = 6.

After establishing that CM derived from LPS-stimulated BV-2 cells did not cause significant negative effects on HT22 cell viability, we investigated whether this CM would have any beneficial potential within oxidative scenarios, including a ferroptotic model. To fulfill this objective, we first investigated the effects of glutamate and *t*-BuOOH on cell viability. Of note, glutamate and *t*-BuOOH have been reported to cause oxidative stress and, in particular, the use of glutamate is a well-established ferroptosis model [11,21,22]. Indeed, in order to investigate the possibility of parallel cell death mechanisms that could be co-acting with glutamate-induced ferroptosis, we pre-treated HT22 cells with 10 µM ferrostatin-1 (classic ferroptosis inhibitor) for 30 min and then exposed them to 5, 10, 15 and 20 mM glutamate for 24 h (Supplementary Figure S2). The impairment of cell viability induced by glutamate was greatly decreased by ferrostatin-1, evidencing ferroptosis as the main mechanism of cell death in our paradigms (Supplementary Figure S2). Additionally, *t*-BuOOH can cause several types of cell death, such as necrosis, apoptosis, necroptosis and parthanatos [23,24,25,26,27,28]. Significant concentration-dependent declines in HT22 cells viability were observed after both glutamate (Figure 3A,B) and *t*-BuOOH (Figure 3C,D) paradigms. The concentrations of 15 mM glutamate and 30 µM *t*-BuOOH, which induced a loss of approximately 50% in the HT22 cell viability after a 24-h treatment, were used in the following studies (Section 2.4).

### 2.4. CM Derived from LPS-Stimulated Microglial Cells Protected against Glutamate-Induced Ferroptosis, but Not against the Oxidative Toxicity Induced by t-BuOOH in HT22 Cells

**Figure 4:**
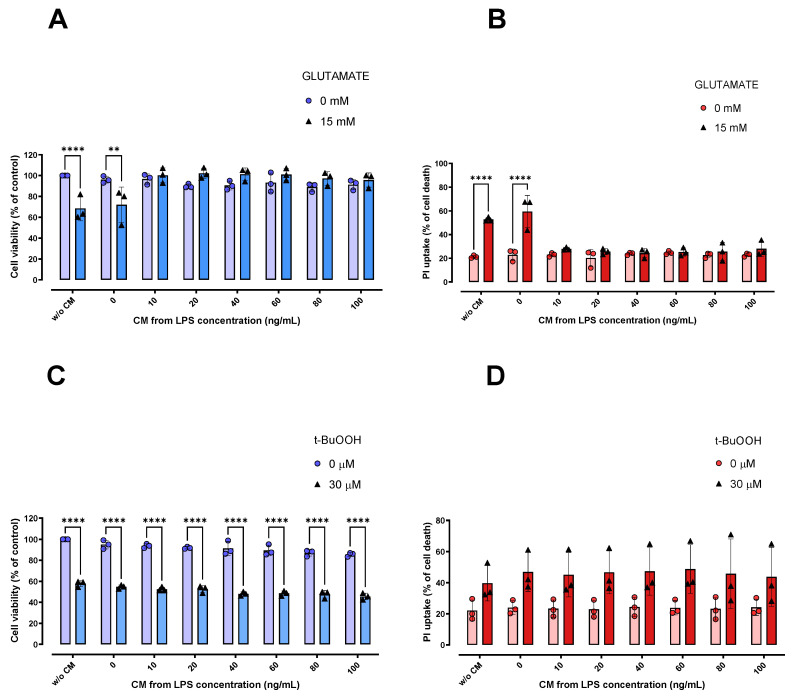
Effects of CM on protection against damage caused by glutamate and *t*-BuOOH in HT22 cells. HT22 cells exposed for 6 h to CM showed protection against cytotoxicity induced by 15 mM glutamate (**A**,**B**). However, this was not observed in the *t*-BuOOH paradigm (**C**). ** *p* < 0.01; **** *p* < 0.0001 compared to the respective controls (cells not treated with glutamate or *t*-BuOOH; *n* = 3). In (**B**,**D**), 100% cell death is represented by cells treated with 0.2% Triton X-100 for 20 min. *n* = 3.

Finally, we evaluated whether the CM derived from LPS-stimulated microglial cells, which has been reported to be rich in (immuno)modulatory mediators [29,30,31], could modulate the oxidative damage induced by both glutamate and *t*-BuOOH in HT22 cells. For this purpose, we pretreated HT22 cells with the CM derived from LPS-stimulated BV-2 cells for 6 h, and then exposed these cells to 15 mM glutamate and 30 µM *t*-BuOOH; these concentrations are capable of inducing approximately 50% loss in cell viability. Of note, HT22 cells pretreated for 6 h with the CM derived from LPS-stimulated BV-2 cells were protected against glutamate-induced ferroptosis (Figure 4A,B), although no significant protection was observed against 30 µM *t*-BuOOH (Figure 4C). As seen in Figure 4A,B, stimuli from 10 to 100 ng/mL of LPS were able to generate a CM that only protected HT22 cells against glutamate-induced cytotoxicity, pointing to a specificity of such protection in a ferroptosis context. It is worth mentioning that the 6-h pretreated with LPS alone was unable to protect HT22 cells against the toxicity induced by 15 mM glutamate (Supplementary Figure S1). This finding indicates the absence of direct effects of LPS in protecting neuronal cells against glutamate oxidative toxicity.

## 3. Discussion

Most of the available scientific literature points to a binary classification of microglial activation, assuming two opposite cell states characterized by a pro-inflammatory (M1) or an anti-inflammatory (M2) profile [32]. Although some lines of evidence indicate that such opposite and polarized states do not represent certain (patho)physiological conditions in the brain [33,34], the M1/M2 nomenclature is commonly accepted and used (including in this manuscript). LPS is a classic inducer of the M1 microglial state, which is usually associated with deleterious effects in neuropathological conditions; the M2 microglial state being desirable [35]. However, our results demonstrated that molecules present in the conditioned medium (CM) derived from a classic M1 microglial activation were able to afford a ferroptosis-resistant phenotype in neurons, indicating that the M1 state is not necessarily linked to neurotoxicity. Conversely, our results indicate that controlled microglial activation towards the M1 state may be beneficial, at least in some specific scenarios. Given the relevance of ferroptosis in neurodegenerative disorders [36], our results represent a relevant finding.

Activated microglia can release a wide range of substances; BV-2 cells treated with 100 ng/mL LPS for 24 h (same concentration/time stimulus used in our work) had a significant increase in NO production and mRNA expression of IL-6, IL-1β, TNF-α and iNOS [37,38]. Of note, a significant increase in TNF-α and IL-6 in CM was also induced with 100 ng/mL LPS in BV-2 cells [30]. Our data suggest that the stimulation caused by these substances mentioned and others derived from microglial activation can induce beneficial effects in neurons. In agreement with our findings, one study reported that the CM derived from LPS-treated mouse-primary microglia presented higher levels of TNF-α, IL-6 and NO, and that such CM was able to increase the survival of midbrain dopaminergic neurons [39]. Of particular importance, this study showed that the physical presence of glial cells is necessary for the induction of neurotoxicity, by comparing neuron-enriched (low percentage of astrocytes and microglia) and neuron-glia cultures (high percentage of astrocytes and microglia, and similar to in vivo conditions) exposed to 1 μg/mL LPS [39]. This observation supports our hypothesis that microglial activation towards M1 is not necessarily deleterious, and suggests that the negative effects attributed to the M1 state may be the result of the complex interaction between glial cells and neurons. Indirectly, this report provides an arguable reason for the low toxicity of CM on HT22 cells observed in our work. In addition, it is noteworthy that the 10 ng/mL LPS concentration was able to increase the nitrite levels by approximately four-fold (from 0.2 to 0.8 micromolar). Thus, it is reasonable to argue that the low concentration of LPS (10 ng/mL) was able to activate microglia, even though at lower rates compared to 100 ng/mL LPS, which was able to increase the nitrite levels by approximately ninety-fold (Figure 1A). In line with this, it was reported that 10 ng/mL LPS induced a significant increase in the expression of inflammatory mediators (M1 markers, such as TNF-α, IL-1β and CD86) [40]. These findings indicate that low microglial activation rates (with stimuli in the order of 10 ng/mL LPS) are sufficient to promote the observed cytoprotection.

Within a pathological and neurodegenerative scenario characterized by chronic microglial activation, there is an undeniable injurious role for pro-inflammatory mediators released by microglia [15]. However, it is reasonable to argue that, under certain conditions, the products of M1 microglial activation may be neuroprotective. For example, TNF-alpha (100 ng/mL) protected cortical neuronal cultures against injury induced by glutamate, N-methyl-d-aspartic acid (NMDA) and deprivation of glucose [41,42]. Similarly, neurons pretreated with IL-1beta (25 ng/mL) exhibited resistance against NMDA-induced cytotoxicity [42]. Finally, SH-SY5Y neuroblastoma cells treated with IL-6 (from 0.025 to 25 ng/mL) resisted the oxidative damage caused by hydrogen peroxide [43]. In addition, IL-6 (10 ng/mL) protected against neurotoxicity induced by 1-methyl-4-phenylpyridinium (MPP+) in dopaminergic neurons [44]. Even though we detected a significant protective effect of LPS-activated BV2 cell-derived conditioned medium in a ferroptosis model—based on the exposure to high (millimolar) concentrations of glutamate in HT22 cells—it is reasonable to hypothesize that the occurrence of such an event is less probable in cells expressing NMDA receptors, such as cultured primary neurons, which undergo excitotoxicity at low (micromolar) glutamate concentrations [45,46,47]. Furthermore, we must not exclude the possibility that a substance of a non-peptide or non-protein nature contributes to the neuroprotective effects. Corroborating this hypothesis, a study reported that the inactivation of proteins by heat and by peptidases/proteases was not able to fully inhibit the protective effect of CM [48]. In this scenario, our study is the first to show that the CM derived from LPS-activated microglial cells has protective effects against ferroptosis in neuronal cells.

Finally, we need to explore the hypotheses for the contrasting effects induced by CM against an oxidative (*t*-BuOOH) and a ferroptotic (glutamate) damage. The pathways by which *t*-BuOOH causes oxidative damage are not fully understood in HT22 cells. It is reported that HT22 cells exposed to 40, 70 or 100 μM *t*-BuOOH exhibit mitochondrial dysfunction and superoxide anion generation [49]. *t*-BuOOH induced necrotic cell death in murine fibroblasts (NIH3T3) and human keratinocytes (HaCaT), an increase in lipid peroxidation and cytosolic ROS sensitive to ferrostatin-1 and liproxstatin-1 was observed [50]. Interestingly, ferroptosis was independently performed of other *t*-BuOOH-induced damage, such as loss of mitochondrial membrane potential and DNA double-strand breaks, thus indicating the participation of cell death type(s) other than ferroptosis [50]. A study with Neuro-2a (mouse neuroblastoma) and SH-SY5Y (human neuroblastoma) demonstrated that treatment with *t*-BuOOH was able to induce cell death via apoptosis [51]. Although ferroptosis has also been reported as a potential type of cell death after *t*-BuOOH exposure—at least in some specific cell types, such as PC12 cells [52]—it is well known that necrosis, apoptosis, necroptosis and parthanatos also represent events resulting from *t*-BuOOH exposure [23,24,25,26,27,28]. Despite glutamate being a classic inducer of excitotoxic cell death in neurons [53,54], such an event is only possible in glutamate N-methyl D-aspartate (NMDA) receptor-containing cells [55]. In fact, the lack of NMDA-receptors in HT22 cells renders such a cell line a classic tool by which to investigate glutamate-induced ferroptosis/oxytosis [56], which is linked to the depletion of glutathione due to the decreased uptake of cystine via the xCT system [36]. Even though our protocols were based on similar intensities of deleterious challenges (an approximate 40% reduction in cell viability after exposures to 15 mM glutamate or 30 µM *t*-BuOOH), the protective effect of activated BV2 cells-derived CM was only observed in the glutamate-based model, pointing to a potential specificity of the protection against ferroptotic challenges.

## 4. Materials and Methods

### 4.1. Chemicals/Reagents

Information concerning the chemicals/reagents used in this work is presented in Table S1, which depicts suppliers and the product codes.

### 4.2. Cell Cultures

The neuronal HT22 cell line was kindly provided by Dr. David Schubert (Salk Institute, La Jolla, CA, USA), and the microglial BV-2 cell line was kindly provided by Professor Licio Augusto Velloso (Universidade Estadual de Campinas, Brazil). The HT22 (mouse hippocampal neurons) and BV-2 (mouse-derived microglia) cell lines were grown in Dubecco’s Modified Eagle Medium (DMEM) supplemented with 10% heat inactivated fetal bovine serum (FBS), penicillin (100 units/mL), streptomycin (100 µg/mL) and glutamine (2 mM) at 37 °C in a humidified atmosphere containing 5% CO_2_. Cells were grown until they were 80–90% confluent and used between the 3rd and 12th passages. All experiments were performed in 96-well plates.

### 4.3. Production of Conditioned Media and Nitric Oxide Assay

BV-2 cells were stimulated with different concentrations of LPS, ranging from 5 to 100 ng/mL, in 96-well plates for 24 h. In order to confirm that such concentrations of LPS were sufficient to induce microglial activation in BV-2 cells, and thus produce a conditioned media (CM), nitric oxide (NO) production was estimated by the measurement of nitrite using the Griess method [30]. Briefly, after 24-h stimulation with LPS, the supernatant of the BV-2 cell culture media was collected and mixed with Griess reagent (0.1% N-1-naphthylenediamine dihydrochloride and 1% sulfanilamide in 1% phosphoric acid), after 10-min incubation at room temperature, the result was analyzed at 543 nm (Tecan Infinite M200, TECAN Instruments, Maennedorf, Switzerland)).

### 4.4. Glutamate and t-BuOOH Exposure Paradigm in HT22 Cells

In order to investigate whether the CM produced by LPS-stimulated BV-2 cells (Section 4.3) would be able to induce a beneficial effect in oxidative scenarios, HT22 cells were exposed to glutamate and *t*-BuOOH, which have been reported to cause oxidative stress, especially glutamate used in ferroptosis models [21,22]. Of note, glutamate is a well-established inducer of ferroptosis [11,57], while *t*-BuOOH can induce different types of cell death, such as necrosis, apoptosis and necroptosis [23,24,25,26,27,28]. HT22 cells were exposed to different concentrations of glutamate (1–20 mM) or *t*-BuOOH (5–100 µM) in 96-well plates for 24 h. After this period, the induction of toxicity was verified by metabolic viability and cell death (Section 4.6 and Section 4.7).

### 4.5. CM Exposure Paradigm in HT22 Cells

HT22 cells were exposed to CM obtained according to item 4.3. Of note, all CM produced was only used in HT22 cells after confirmation of microglial activation (nitrite production) in most groups. Briefly, HT22 cells were seeded in 96-well plates for 24 h, then half (50 µL) of their culture medium was removed and replaced with 50 µL of CM for 6 h. After this period, HT22 cells were exposed for 18 h to 15 mM glutamate or 30 µM *t*-BuOOH.

### 4.6. Cell Metabolic Viability Assay

The metabolic cell viability of HT22 and BV-2 cells was evaluated through their ability to reduce 3-(4,5-dimethylthiazol-2-yl)-2,5-diphenyl-tetrazolium bromide (MTT), which was quantified as previously described [58]. After the cells were exposed to LPS, CM, glutamate or *t*-BuOOH in 96-well plates, their culture medium was removed and an MTT solution was added, followed by 1-h incubation at 37 °C. Afterwards, the wells were emptied and the formazan product was dissolved in dimethyl sulfoxide (DMSO), and the results were quantified at 540 nm (Tecan Infinite M200).

### 4.7. Cell Death Assay

Cell death assay was performed based on the incorporation and binding of propidium iodide (PI) to the DNA, producing red fluorescence in cells with damaged plasma membranes [59]. After the cells were exposed to LPS, CM, glutamate or *t*-BuOOH in 96-well plates, 147 μg/mL propidium iodide (PI) was added to the cells, and then incubated for 20 min at 37 °C in the dark. Fluorescence was quantified at 535 nm excitation and 617 nm emission (Tecan Infinite M200, TECAN Instruments, Maennedorf, Switzerland).).

### 4.8. Statistical Analysis

Initially, Grubbs’ test, also called the ESD method (extreme studentized deviate), was performed to detect and exclude significant outliers. Data were subjected to D’Agostino and Pearson’s normality test. In vitro data were analyzed using one-way or two-way analysis of variance (ANOVA), followed by Tukey’s post hoc test. Graphics and ANOVAs were performed using Graph-Pad PRISM^®^ software version 7.0a (GraphPad Software, San Diego, CA, USA). The results were expressed as mean ± SD and significance was considered when *p* < 0.05.

## 5. Conclusions

In summary, our results show that substances derived from M1 microglial activation are not necessarily toxic, adding evidence to the debate against the dichotomous classification with pre-established functions for M1 and M2 (neurotoxic and neuroprotective, respectively). Taken together, our data reveal an unexplored therapeutic possibility, i.e., the modulation of ferroptosis through microglial activation products, shedding light on a new event related to beneficial communication between neurons and microglia.

## Data Availability

All data in the current study are available upon reasonable request.

## Supplementary Materials

The supporting information can be downloaded at: https://www.mdpi.com/article/10.3390/ijms24032910/s1.
