# Supplementary material for: LPS-Activated Microglial Cell-Derived Conditioned Medium Protects HT22 Neuronal Cells against Glutamate-Induced Ferroptosis
Source: Int J Mol Sci. 2023 Feb 2;24(3):2910. doi: 10.3390/ijms24032910 (PMC9917809; doi:10.3390/ijms24032910)
Supplement: Supplementary file 1 [file ijms-24-02910-s001.zip › ijms-2086969-supplementary.pdf]

Supplementary Materials

Supplementary Table S1: Chemicals and reagents used in the study.

**Chemicals/reagents**	**Supplier**	**Code**
**3-(4,5-Dimethyl-2-thiazolyl)-2,5-diphenyl-2H-tetrazolium bromide (MTT)**	**Sigma-Aldrich**	**M2128**
**Antibiotic-Antimycotic (100X)**	**Thermo Fisher**	**15240062**
**Dimethyl sulfoxide (DMSO)**	**Sigma-Aldrich**	**472301; D2650**
**Dulbecco's modified Eagle's medium (DMEM)**	**Thermo Fisher**	**12100046**
**Fetal bovine serum (FBS)**	**Thermo Fisher**	**16140071**
**Hanks' Balanced Salt solution (HBSS)**	**Sigma-Aldrich**	**H8264**
**L-Glutamic acid**	**Sigma-Aldrich**	**G1251**
**Lipopolysaccharides from *Escherichia coli* O111:B4**	**Sigma-Aldrich**	**L2630**
***N*-(1-Naphthyl)ethylenediamine dihydrochloride**	**Sigma-Aldrich**	**222488**
**Propidium iodide (PI)**	**Sigma-Aldrich**	**P4170**
**Sulfanilamide**	**Sigma-Aldrich**	**S9251**
***tert-Butyl hydroperoxide* (t-BuOOH)**	**Sigma-Aldrich**	**458139**
**Triton X-100**	**Sigma-Aldrich**	**X100**
**Trypsin-EDTA**	**Thermo Fisher**	**15400054**

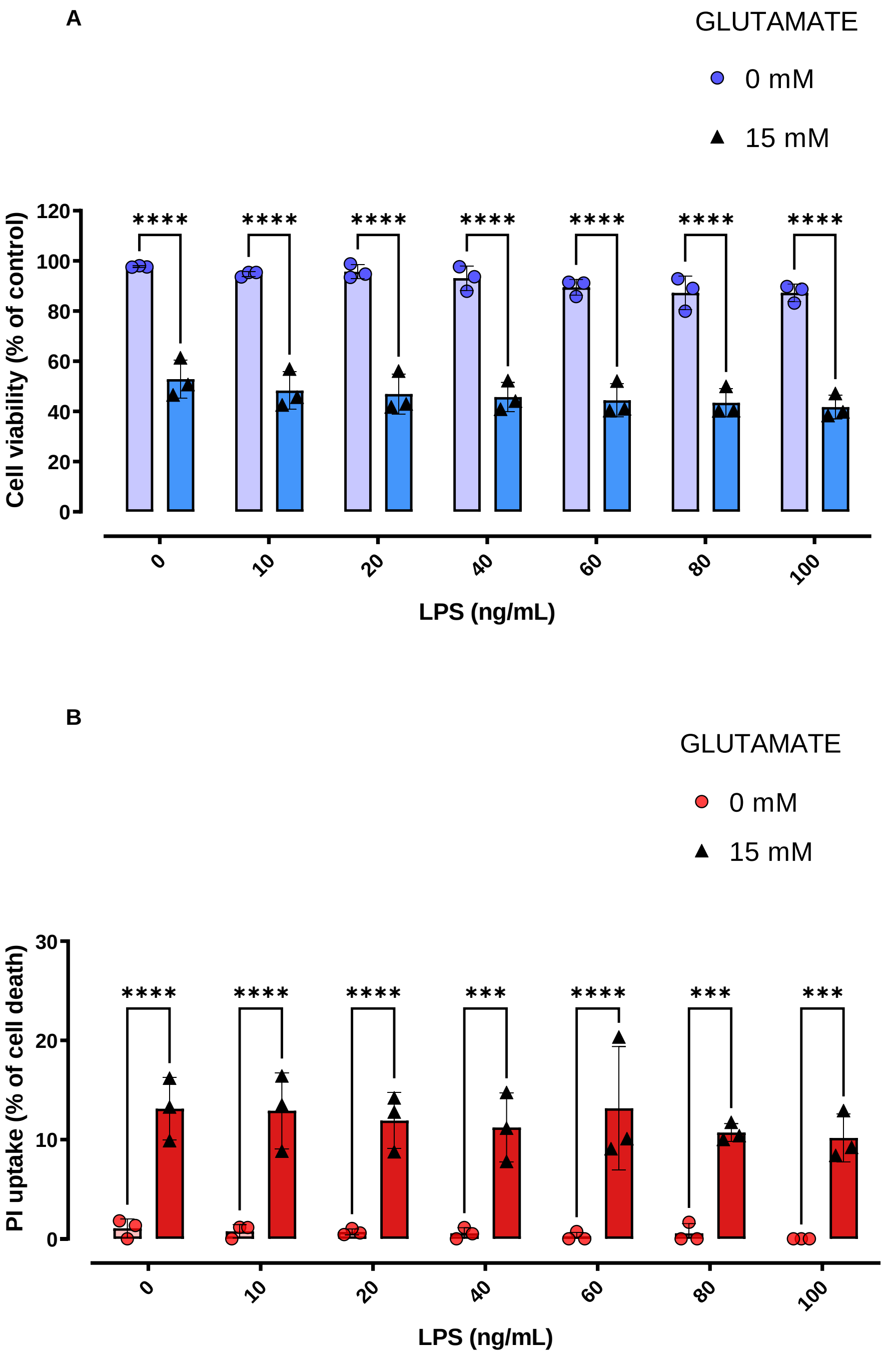

Supplementary Figure S1. LPS did not induce protection against 15 mM glutamate. In order to investigate whether LPS would be able to generate a protective response against glutamate-induced ferroptosis, HT22 cells were exposed for 6 h (item 4.5) to different concentrations of LPS and then to 15 mM glutamate for 18 h. The figure shows significant impairment of metabolic cell viability (A - MTT assay) and increased cell death (B - PI assay) induced by 15 mM glutamate. These results were not significantly altered by pre-exposure to LPS (10-100 ng/mL), i.e., no protective effects against 15 mM glutamate were observed. In B, 100% cell death is represented by cells treated with 0.2% Triton X-100 for 20 minutes. ***$p < 0.001$; ****$p < 0.0001$ compared to control group (untreated cells). n=3.

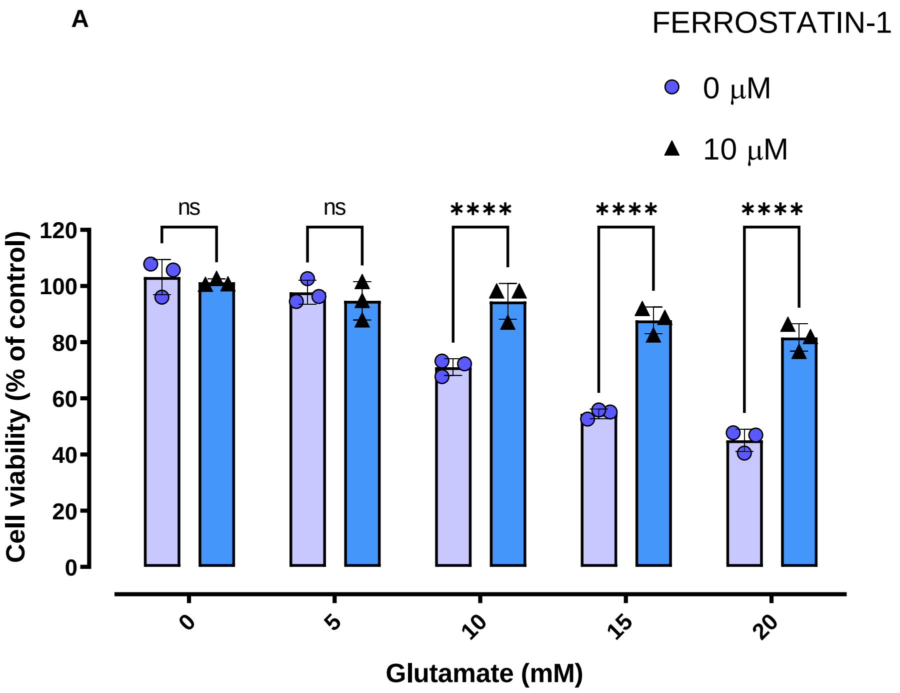

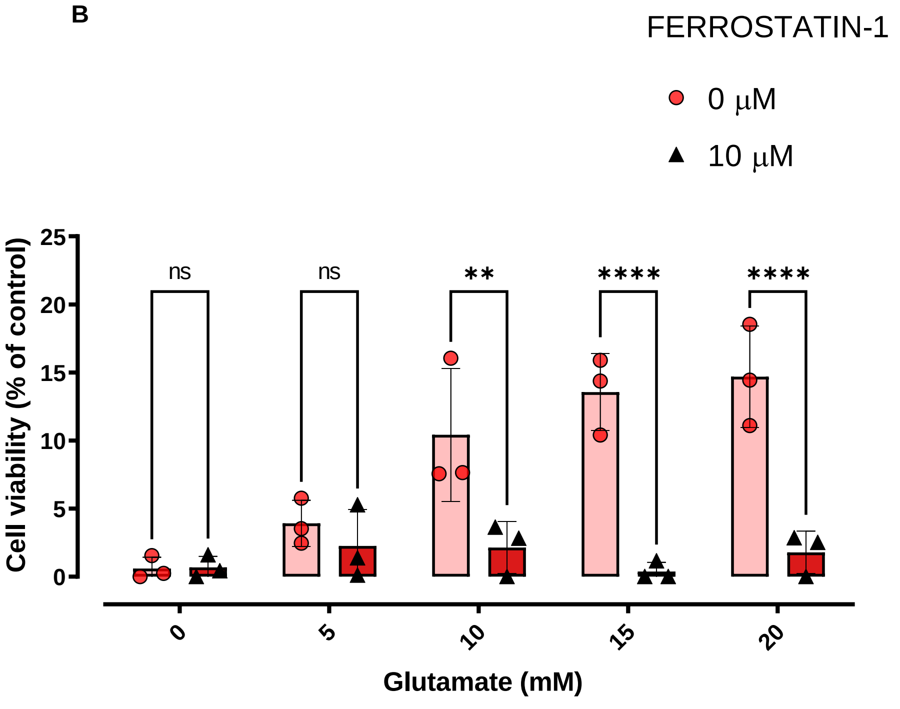

Supplementary Figure S2. Ferrostatin-1 protected against glutamate-induced cell death. In order to investigate parallel cell death mechanisms that could be co-acting with glutamate-induced ferroptosis, HT22 cells were pre-treated with 10 µM ferrostatin-1 (classic ferroptosis inhibitor) for 30 min and then exposed to 5, 10, 15 and 20 mM glutamate for 24 h. The figure shows significant impairment of metabolic cell viability (A - MTT assay) and increased cell death (B - PI assay) induced by 10, 15 and 20 mM glutamate. These effects were

greatly decreased by pre-treatment with ferrostatin-1, evidencing ferroptosis as the main mechanism of cell death in our paradigms. In B, 100% cell death is represented by cells treated with 0.2% Triton X-100 for 20 minutes. ns: not significant; $^{**}p < 0.01$; $^{****}p < 0.0001$ compared to control group (untreated cells). n=3.
